# The relationship between ankle landing kinematics, isokinetic strength, muscle activity, and the prevalence of lower extremity injuries in university-level netball players during a single season

**DOI:** 10.17159/2078-516X/2024/v36i1a16918

**Published:** 2024-05-15

**Authors:** TT Jolingana-Seoka, HV Hammill, Y Willemse, M Kramer

**Affiliations:** 1 Physical Activity, Sport and Recreation (PhASRec), Faculty of Health Sciences, North-West University (NWU), Potchefstroom, South Africa; 2 Centre for Health and Human Performance (CHHP), Faculty of Health Sciences, North-West University (NWU), Potchefstroom, South Africa

**Keywords:** dynamometer, EMG, motion analysis, multidirectional, team-sport

## Abstract

**Background:**

Safe landing in netball is fundamental. Research on the biomechanics of multidirectional landings is lacking, especially among netball players. Furthermore, few studies reporting the associations between ankle kinematics, isokinetic ankle muscle strength, muscle activities, and injury prevalence in South African netball have been undertaken.

**Objectives:**

To determine the relationships between ankle kinematics, kinetics, isokinetic strength, and muscle activity during jump-landing tasks, as well as the prevalence of lower extremity injuries in university-level netball players during a single season.

**Methods:**

This cross-sectional repeated-measure study consisted of ten university-level female netball players. The injury prevalence data was collected during the 2022 netball season. The ankle muscle activity, kinematic, and kinetic data were collected during multidirectional single-leg landing and muscle strength was collected from plantar- and dorsiflexion trials.

**Results:**

Netball players’ ankle strength was generally below average. There was evidence of negative correlations between the ankle range of motion (ROM), isokinetic strength, and muscle activity amplitudes. A lack of evidence prevented the conclusion that lower extremity dominance predisposed players to injury, and that any specific body part was more likely to be injured among netball players.

**Conclusion:**

Landing forces and muscle activity are direction-dependent, especially for the dominant limb. Lower extremity strength and neuromuscular control (NMC) across multiple jump-landing directions should be an area of focus for female netball players.

In netball, an athlete’s ankle joint can absorb landing forces of three to five times their body weight (BW) from a single jump, excluding forces resulting from running and shuffling across the court. ^[1]^ Typically, such large forces are unilaterally directed as netball players tend to land on a single leg approximately 65% of the time during their gameplay and practices, which could partially explain the vulnerability of the lower extremity to injury. ^[1]^ Furthermore, landing forces vary considerably depending on the landing direction, the task performed, foot placement, jumping distance, and landing strategy. ^[1–4]^

Given these demands, the ankle is reliant on adequate joint motion, sufficient muscle strength and proper muscle recruitment to attenuate the associated landing forces, guard against injury, and enhance performance.^[1]^ Given the multidirectional nature of landing in netball, it is perhaps not surprising that during competition, approximately 84% of all injuries are sustained at the ankle, specifically to the lateral ligament complex. ^[1,5]^ Analyses of landing ability in netball players should, therefore be multifaceted and account for (i) movement *direction* (e.g., lateral, diagonal, forward etc.) and *orientation* (e.g., inward vs. outward), (ii) the magnitude of landing forces, and (iii) whether ankle strength and muscle activity are associated with landing direction and/or orientation. ^[4]^ Evaluations of muscle function can provide the basis for interventions that seek to improve landing performance and potentially provide insights into muscle contributions during the downward motion upon landing.^[6]^ The kinematics, kinetics, and dynamic postural stability associated with multidirectional landing are likely to be varied, especially during single-leg manoeuvres, due to the large demands placed on the joints of the lower extremities. ^[7–9]^ Neuromuscular deficits in the lower extremities may influence dynamic postural control and stability during landing, resulting in reduced time to reach a stable position upon landing and subsequently increasing the risk of lower extremity injury.^[1]^ Furthermore, neuromuscular control is best evaluated by measuring muscle activities of the muscles around the joint, in addition to dynamic stability, to address preparatory (feedforward) and reactive (feedback) neuromuscular mechanisms required for foot positioning and for the maintenance of better joint stability. ^[10]^ Additionally, strength asymmetry in the ankle joint musculature is one of the identified risk factors for injury in sports and may lead to alterations in neuromuscular control. ^[1,11]^ Sufficient lower extremity strength has been associated with improved postural stability, adequate joint motion, and lower ground reaction forces (GRF). ^[8]^ However, ankle isokinetic strength is under-researched as compared to knee isokinetic strength. As a result, only a limited number of studies have researched normative data for ankle isokinetic strength in different sporting populations, or the transferability of isokinetic strength to dynamic multidirectional landing tasks.

Given the current gaps in the literature, it is presently unclear whether associations between lower extremity muscle activity, isokinetic strength, and multidirectional landing kinematics are evident, especially among netball players. Subsequently, the primary objectives of the present study were to determine (i) differences in electromyogram (EMG) amplitude as a function of the landing leg, landing direction, or muscle group, (ii) the relationship between ankle kinematics, muscle activity, and isokinetic strength, and (iii) the injury prevalence among university-level netball players in a single netball season. We hypothesised that the landing direction would significantly influence EMG amplitude and that there would be a significantly negative correlation between ankle kinematics, muscle activity, and isokinetic strength. We further hypothesised that there would be a high prevalence of injuries in university-level netball players during one netball season. It is important to provide information on landing kinematics, ankle muscular strength, and muscle activity to determine which parameters could be useful for improving safe landing kinematics, designing injury prevention and ankle strengthening programmes, and helping coaches make informed decisions about athletes’ safe return to play.

## Methods

### Participants

An optimal sample size of 13 female participants was calculated *a priori* for a repeated measures study design using the following input parameters for sufficient statistical power: alpha = 0.05; power = 0.80; repeated measures = 5; correlation among repeated measures = 0.70. A total of 15 participants volunteered for testing, and a final sample of 10 was retained for analysis (injured: n=3; technical error: n = 2) in the cross-sectional repeated measures study. Ethical approval was obtained from the Faculty of Health Science, Human Research Ethics Committee of the North-West University (NWU) (NWU-00154-21-S1) and NWU Research Data Gatekeeper Committee (NWU-GK-21-071). Voluntary written informed consent was obtained from participants before testing.

### Testing

A week before testing, an information session was held to explain the purpose and possible risks of the study, as well as the procedures involved in the testing protocol. The participants’ demographic information was collected by utilising an electronic demographic and injury information questionnaire at the beginning of the netball season. Feedback on injury occurrence was electronically monitored, with participants filling in the injury information questionnaire every second week until the end of the season. Participants were encouraged to report an injury as soon as it occurred to prevent any loss of information concerning injury occurrence incidences.

#### Ankle isokinetic strength

Before isokinetic strength testing, participants completed a five-minute warm-up on a Wattbike cycle ergometer (WattbikePro, Technogym, USA), at a cadence of 75 revolutions per minute (RPM), with the wind and magnetic resistance set at 1. Dynamic stretches for the major muscle groups of the lower extremity followed the warm-up. Participants were then asked to sit comfortably (modified position) on the isokinetic dynamometer (Cybex Humac Norm model 770 Cybex, division of Lumex Inc., Ronkonkoma, NY, USA) as per user guidelines. Participants were further instructed to keep their arms across their chest throughout the test to eliminate force transfer. Participants were given a standardised trial of three repetitions as a warmup to familiarise them with the testing process. Strength of the ankle plantar- and dorsiflexors was evaluated by completing five repetitions during a 60 degrees per second (deg/s) concentric/concentric (CON/CON) protocol, whereas muscle endurance was evaluated during a 120 deg/s (CON/CON) protocol. Participants rested for ten seconds between each trial and 180 seconds between sets. Testing was initiated on the dominant limb (DL), then repeated on the non-dominant limb (NDL).

#### Muscle activity

All EMG data were recorded at 2000 Hertz (Hz) using an eight-channel system (Noraxon Ultium, USA, Scottsdale, AZ). Electrodes were placed on the tibialis anterior (TA), on the head of the medial gastrocnemius (MG), and on the peroneus brevis (PB) in accordance with SENIAM guidelines (Surface ElectroMyography for the Non-Invasive Assessment of Muscles). All EMG data were exported to MATLAB for processing. Firstly, raw data were detrended (to remove any potential drift), then filtered using a second-order, zero-lag Butterworth filter with a bandpass of 10–500 Hz to remove unwanted noise and enhance the signal-to-noise ratio. The processed and filtered data were then rectified and smoothed using a moving average of 500 ms^−1^. The mean EMG activation amplitude within a specific region of interest was defined as ranging from 100 ms^−1^ before landing (pre-landing phase; see Force plate signals for the definition of landing), until the end of the eccentric phase (post-landing phase; see force plate signals for the definition of ‘end of eccentric phase’).

#### Landing kinematics and kinetics

Kinematic data of the lower extremities were recorded at 2000 Hz during the jump-landing tasks using an eight-camera motion capture system (Oqus 300+, Qualisys, Goteborg, Sweden). Before data collection, the capture volume was fully calibrated using a calibration wand and L-frame to achieve sub-millimetre precision. The lower extremity CAST marker set was used whereby markers were attached bilaterally on the anterior superior iliac spine (ASIS), the posterior superior iliac spine (PSIS), the greater trochanter, the medial and lateral femoral epicondyles, the medial and lateral malleoli, calcaneus, as well as on the first, second and fifth metatarsal heads of DL and NDL lower extremities.^[4]^ After static calibration, the markers on the femoral condyles, malleoli, and greater trochanters were removed for execution of dynamic trials.

Participants performed single-leg jumps bilaterally from a 0.30 m box onto a force plate (AMTI, Gen5, Advanced Medical Technology, MA, USA) located 0.70 m from the box. The participants jumped in the following directions: straight jump (SJ), diagonal inside (DI), diagonal outside (DO), lateral inside (LI) and lateral outside (LO), during an eight-second capture period.^[4]^ Participants were required to perform five successful jumps per direction per leg, resulting in 25 jumps per lower extremity. Unsuccessful jumps (i.e., instances where participants landed bilaterally or lost balance) were discarded from the analysis and the trial was repeated. All force data were recorded at 2000 Hz and exported to MATLAB for processing (The MathWorks Inc. (2022). MATLAB version: 9.13.0 (R2022b), Natick, Massachusetts: The MathWorks Inc. URL: https://www.mathworks.com). Raw force data were used to establish key phases such as *landing* (when the vertical force exceeds a 30 Newton (N) threshold), *end-of-eccentric phase* (minimum in the vertical centre of mass (CoM) displacement after landing determined by inverse kinetics), and *time-tostabilization* (time point when the force vector remains within body weight (BW) ± 5 SD following landing).

Finally, all marker data were exported to Visual3D (C-Motion, version 2022.08, USA) to extract tri-planar lower extremity kinematics. Kinematic data were filtered using a low-pass, bi-directional Butterworth filter with a cut-off frequency of 6–12 Hz.

### Statistical analysis

The normality of data were evaluated using the Shapiro-Wilk test, with deviations from normality being accepted at p < 0.05. To evaluate differences in EMG amplitude, a three-way repeated-measures analysis of variance (ANOVA) was utilised. The repeated measure factors included the *landing leg* (two levels: DL vs. NDL), *landing direction* (five levels: DI, DO, LI, LO, SJ), and *muscle group* (three levels: TA, MG, PB). For instances where significant differences were present, the Tukey correction was used to adjust for multiple comparisons. Partial-eta-squared (η^2^p) was used as the standardised effects size and was qualitatively interpreted as follows: small: η^2^p = 0.01; medium: η^2^p = 0.06; large: η^2^p = 0.14.^[12]^ Differences in relative peak landing forces were evaluated using a Friedman one-way repeated measures ANOVA where Kendall’s coefficient of concordance (Wkendalls) was used as the standardised effect size. The standardised effect size was qualitatively interpreted as: slight agreement: 0.00 – 0.20; fair agreement: 0.20 – 0.40; moderate agreement: 0.40 – 0.60; substantial agreement: 0.60 – 0.80; almost perfect agreement: > 0.80. ^[13]^ For those instances where significant differences were present, the Holm correction was used to adjust for multiple comparisons.

A correlation analysis between ankle ROM, isokinetic strength, and muscle activities was completed using Spearman’s rank correlation coefficient with a Holm correction to adjust for multiple comparisons. The magnitude of the coefficients was qualitatively interpreted in absolute terms as follows: negligible: r = 0.00 – 0.10; weak: r = 0.11 – 0.39; moderate: r = 0.40 – 0.69; strong: r = 0.70 – 0.89; very strong: r = 0.90 – 1.00.^[14]^ Differences in injury prevalence across the joints and lower extremities were evaluated using the Chi-squared goodness of fit test (χ^2^gof). ^[15]^ For all instances, the threshold for statistical significance was set at p ≤ 0.05. All statistical analyses were completed using the R-programming language ^[16]^ and Jamovi (The Jamovi project (2023), *v*ersion 2.2.5.0, [Computer Software], URL https://www.jamovi.org).

## Results

**Fig. 1:**
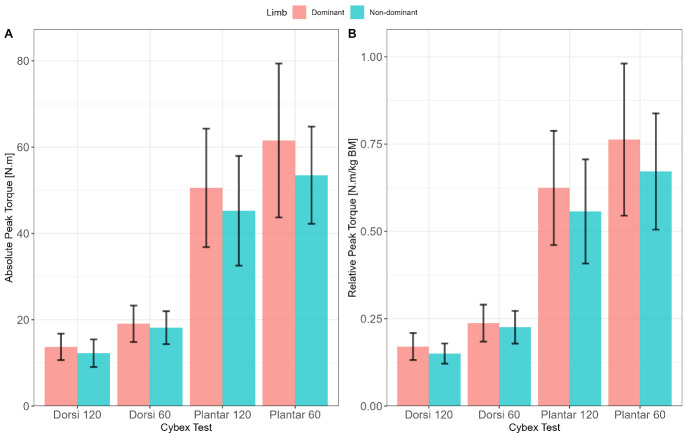
Absolute and Relative Peak Torques. Panel A: Absolute Peak Torque for ankle dorsi- and plantar flexors at 60 deg/s and 120 deg/s as a function of the dominant (red) and non-dominant (blue) limbs. Panel B: Relative Peak Torque for ankle dorsi- and plantar flexors at 60 deg/s and 120 deg/s as a function of the dominant (red) and non-dominant (blue) limbs.

**Fig. 2:**
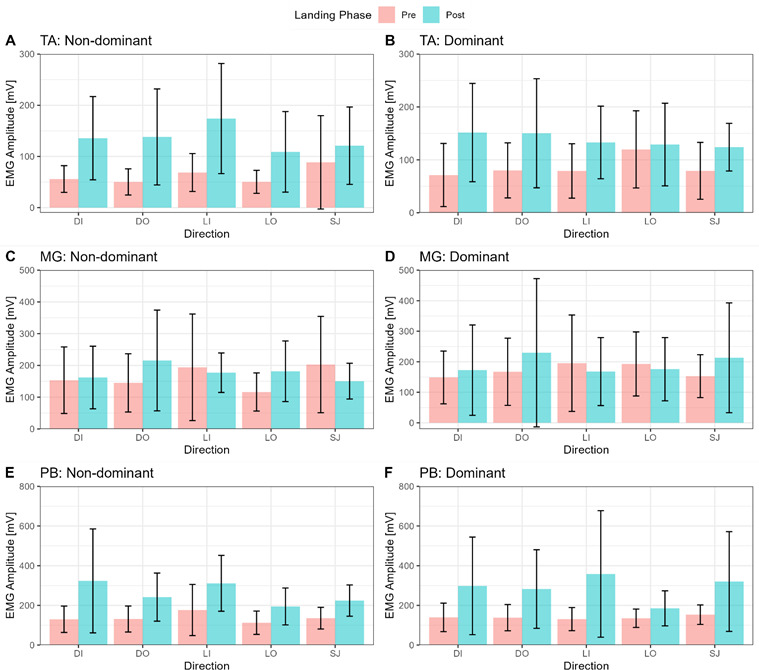
Mean muscle activation amplitude pre- and post-landing for the TA (tibialis anterior), MG (medial gastrocnemius) and PB (peroneus brevis) across multiple directions. mV, millivolts; EMG, electromyogram; DI, diagonal inside; DO, diagonal outside; LI, lateral inside; LO, lateral outside; SJ, straight jump.

A total of 10 female netball players were part of the investigation (age: 21.2 ± 1.4 years; stature: 1.77 ± 0.74 m; body mass: 80.3 ± 12.3 kg). The mean (m) and standard deviation (SD) data for absolute and relative ankle peak torques (PT) (dorsiand plantarflexion at 60 deg/s and 120 deg/s) are presented in Figure 1. Overall, the dominant lower extremity produced greater absolute PT during dorsi- and plantarflexion at 60 deg/s and 120 deg/sec, respectively, than the non-dominant lower extremity (Figure 1A). Similar trend is observed in relative PT (Figure 1B). Additionally, slower speed (60 deg/s) produced higher PT compared to higher speed (120 deg/s). The muscle activation amplitude of the TA, MG, and PB varied considerably pre- and post-landing phase between the DL and NDL as a function of landing direction (Figure 2A–F).

The inferential results regarding differences in EMG amplitude for each leg, landing direction, and muscle group are shown in Table 1. Statistically significant differences were evident for EMG amplitude between muscle groups (F (1,9) = 0.93, p < 0.001, η^2^p = 0.65). Post-hoc comparisons indicated significant differences between TA and PB (Mdiff = −137.46 mV, p = 0.004), between MG and PB (Mdiff = −89.47 mV, p < 0.001), but not between TA and MG (Mdiff 203 = −47.99, p = 0.170).

The results for the three-way repeated measures ANOVA are shown in Table 1.

**Fig. 3:**
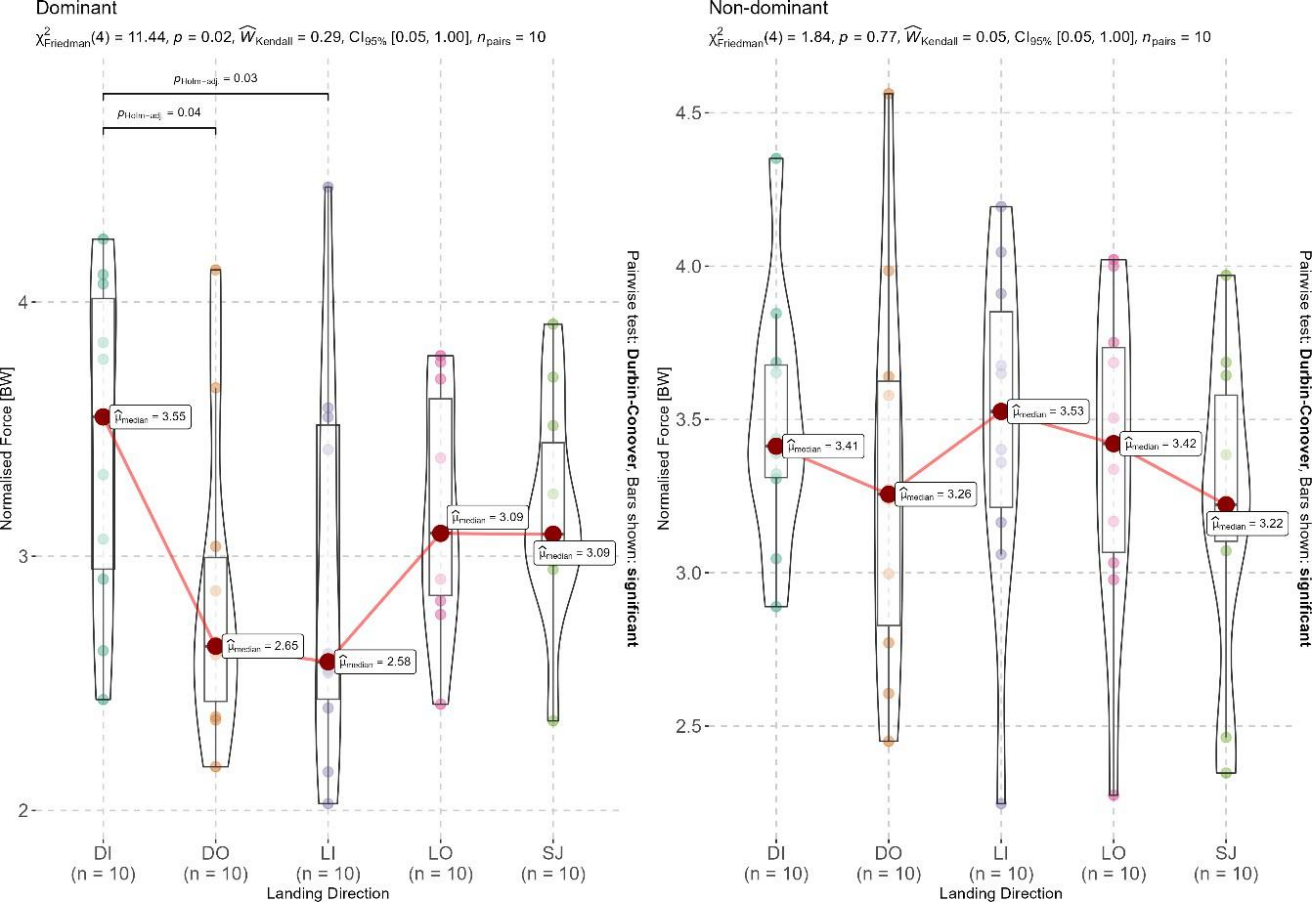
Relative peak landing forces as a function of lower extremity dominance and landing direction. DI, diagonal inside; DO, diagonal outside; LI, lateral inside; LO, lateral outside; SJ, straight jump; BWs, body weights.

Results for the normalised peak landing forces as a function of landing leg and direction are shown in Figure 3. In general, the mean landing forces for the NDL and DL were 3.37 ± 0.53 BWs and 3.12 ± 0.62 BWs respectively. More specifically, significant differences were evident between the DI and DO directions (p = 0.04) and between DI and LI directions (p = 0.03) but not for the other directions for the DL. No direction-specific differences were evident between landing directions for the NDL.

**Fig. 4:**
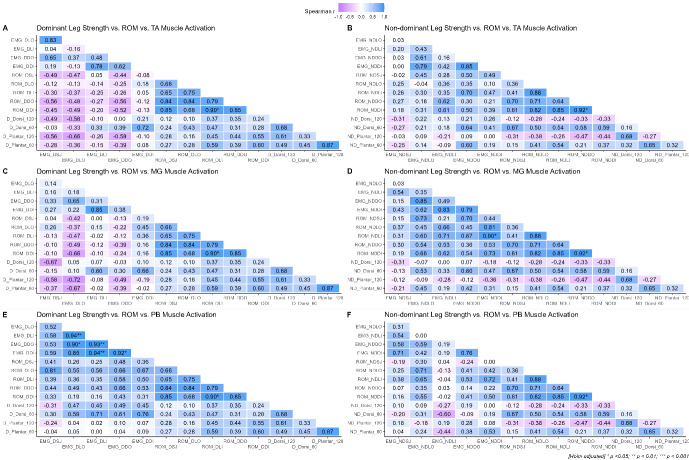
Correlation analysis between ankle range of motion (ROM), relative peak ankle isokinetic torque and mean pre-landing electromyogram (EMG) amplitude for each muscle group. Panels A–B: tibialis anterior (TA) for the DL and NDL, Panels C–D: medial gastrocnemius (MG) for the DL and NDL, and Panels E–F: peroneus brevis (PB) for the DL and NDL. Correlation coefficients are coloured such that negative associations are purple-shifted and positive associations are blue-shifted. The colour intensity provides information related to the magnitude of the correlation (i.e. darker = stronger; lighter = weaker). LO, lateral outside; LI, lateral inside; DO, diagonal outside; DI, diagonal inside; SJ, straight jump; Dorsi, dorsiflexors; Plantar, plantar flexors; 60, 60 deg/s; 120, 120 deg/s.

The correlations between ankle ROM, ankle strength and muscle activation amplitude of the TA, MG, and PB, prior to landing for both the DL and NDL are shown in Figure 4A–F.

In general, distinctive patterns emerge across the different muscle groups prior to landing. Firstly, for the TA of the NDL side, moderate positive associations are evident for the relationship between mean EMG amplitude and ROM across all directions whereas weak-to-moderate negative associations are evident for the DL. The converse is true for the associations between muscle strength and ROM in that moderate-to-strong negative correlations are evident for the DL side, and negligible-to-moderate correlations are evident for the DL side. Finally, the correlations between isokinetic muscle strength and EMG amplitude exhibit negligible-to-moderate negative relationships for both the NDL and DL, implying that the stronger the muscle tends to be, the lower the EMG amplitude. The same trends hold true for the MG and PB muscle groups.

**Fig. 5:**
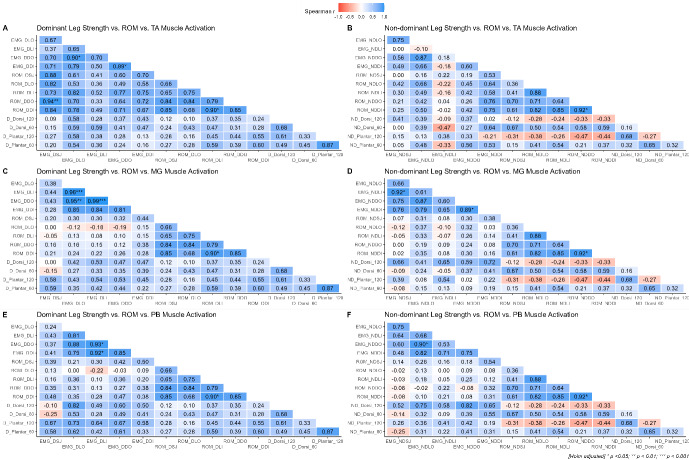
Correlation analysis between ankle range of motion (ROM), relative peak ankle isokinetic torque and mean post-landing electromyogram (EMG) amplitude for the tibialis anterior (TA), medial gastrocnemius (MG) and peroneus brevis (PB) for non-dominant (ND) and dominant (D) lower extremities. Correlation coefficients are coloured such that negative associations are red-shifted and positive associations are blue-shifted. The colour intensity provides information related to the magnitude of the correlation (i.e. darker = stronger; lighter = weaker). LO, lateral outside; LI, lateral inside; DO, diagonal outside; DI, diagonal inside; SJ, straight jump; Dorsi, dorsiflexors; Plantar, plantar flexors; 60, 60 deg/s; 120, 120 deg/s.

The correlations between ankle ROM, ankle isokinetic strength and muscle activation amplitude of the TA, MG, and PB during the eccentric phase of landing for both the DL and NDL are shown in Figure 5A–F. Distinctive patterns in the correlation data were evident, whereby small-to-large positive associations were observed between EMG amplitude and ankle ROM for all muscle groups of the DL. Furthermore, small-to-moderate negative associations were apparent between muscle strength and ROM for the NDL across all landing directions, whereas negligible-to-moderate positive associations were evident for the NDL. Generally, small-to-moderate positive relationships between muscle strength and EMG amplitude are apparent for all muscle groups, except for the TA of the NDL where negligible-to-moderate negative were manifest.

Finally, differences in injury prevalence as a function of body part and lower extremity dominance across the netball season were reported in the current study. Injuries to the ankle joint dominated (30%), followed by injuries to the foot (20%), back (20%), knee (10%), hip (10%), and calf (10%). Due to the low number of injuries sustained over the season, there were no statistically significant differences in terms of the body part most likely to be injured (χ2gof (5) = 2.00, p = 0.850) or the lower extremity most likely to be injured (χ2gof (1) = 0.00, p = 1.00).

## Discussion

The ability to land safely and effectively depends on adequate strength and neuromuscular coordination of the muscles associated with the lower extremities. ^[1,6]^ More specifically, during the eccentric phase of single-leg landing, the quadriceps, soleus, and gluteus muscles tend to contribute the greatest amount of mechanical work to arrest the CoM when landing. ^[6,17]^ The present study showed that during the eccentric phase of landing, the MG and PB exhibited high activation amplitudes but that these were not significantly different across landing directions (F (4.36) = 1.03, p = 0.406, η^2^p = 0.10). It is therefore plausible that the neuromuscular strategies associated with single-leg landing were similar across landing directions and largely independent of lower extremity dominance, at least within the sample analysed. Interestingly, the strength of the dorsi- and plantar flexors in the present sample of netball players were below the expected values of similarly aged netball individuals, ^[18]^ yet they were able to comfortably withstand the moderately large landing forces experienced during single-leg landing.

Apart from limitations to the Cybex testing, it is possible that despite lacking the expected ankle isokinetic strength, netball players are proficient enough at landing, such that they can effectively mitigate the impact associated with landing. Moreover, it is important to note that landing is reliant on adequate contributions from proximal joints to the ankle (e.g., knee and hip), and it is therefore also possible that netball players may have a more knee or hip-dominant landing strategy, which was not evaluated in the present study.

Although the torques and speeds of isokinetic testing do not match netball-specific demands, isokinetic testing remains the gold standard for evaluating muscle strength after injury. Given that there is currently a lack of pre-injury strength data across almost all major joints, such information is seriously needed to better contextualise post-injury strength and enhance return-to-play decision-making. Adequate ankle ROM during drop landing is known to attenuate the GRF by facilitating energy absorption. ^[19]^ As drop height and velocity increase, individuals tend to extend the knee and ankle joints to a greater extent to augment the time over which forces can be dissipated.^[19]^ The extent to which ankle joint isokinetic strength is coupled with ankle ROM during landing has, however, not been previously investigated to the knowledge of the authors at the time of the present study.

The present study showed that, at least within the nondominant limbs of netball players, there were substantial negative correlations between ankle plantarflexor strength at 120 deg/s and ROM during landing (e.g., r = −0.57 to −0.79). The associations appear to be dependent on the landing direction, and tend to indicate that the greater the level of plantarflexor strength, the lower the ankle ROM required during landing. Such an association was not apparent for the dominant limb where substantially smaller associations were present (i.e., r = −0.13 to 0.42). The joint motion and coordination of various lower extremity muscle activities allow for a ‘better’ landing by effectively reducing the impact forces associated with landing. ^[7]^ The muscle activities of TA, MG, and PB are often reported in studies investigating the landing action (i.e., drop landing) as they play a crucial role in controlling the ROM of the ankle joint at touchdown. ^[7,20]^ The primary function of TA is to dorsiflex the foot, the PB prevents the ankle from going to excessive inversion and supination, while MG plantarflexes the foot and is responsible for deceleration during landing.^[7,20]^ Inhibition of these muscles may be a clear indication of neuromuscular deficits and could lead to reduced stability and injuries to the ankle. Inhibition of the peroneal musculature, specifically, has been identified as a risk factor for instability and injury in players.^[11]^ The reduced activation of the PB could lead to an increased inversion angle at initial ground contact, which can subsequently lead to ankle inversion sprain and ankle instability. ^[20]^ Although the literature identifies the ankle joint as being predisposed to injury, there is limited research on injury prevalence in South African university-level netball players. ^[5]^ The results of the present study reported injury to the ankle joint as the most prevalent (30%) which concurs with the findings of other authors. ^[5]^ In their study over two consecutive netball seasons, Sinclair et al. reported an ankle joint injury prevalence of 38% among similarly aged elite senior netball players in the Free State province.^[5]^ Furthermore, most biomechanical studies investigating injury risk and landing manoeuvres seldom report risk of injury between bilateral ankle joints during landing, as only the data pertaining to the DL are reported. ^[7]^ It is therefore important to provide information related to bilateral ankle biomechanics during multidirectional, single-leg landing which the present study sought to address. Limitations associated with the current study include the following: muscle torque was evaluated solely in the sagittal plane, whereby future investigations should incorporate the frontal- and/or transverse plane ankle torques to provide a more holistic understanding of potential ankle injury mechanisms. Eccentric muscle strength testing of the ankle was not evaluated in the present study and should be included to complement jump-landing manoeuvres. A larger sample size across a more heterogenous group of individuals should enhance the generalisability of the findings and provide more precise point estimates for each parameter evaluated. A larger sample evaluated over a longer timeframe (e.g. more than a single season) would provide a greater opportunity to evaluate the effects of interventions on specific injuries, as well as provide greater insights related to injury rates.

### Practical applications

Strength and conditioning coaches can use the tests outlined in the present study as baseline measurements for designing a training program targeting strength, NMC, evaluating landing technique and monitoring the effectiveness of an intervention program. This information can form part of testing battery for netball players to identify any predisposing deficits so as to mitigate the risk of injury/re-injury to the lower extremities. By doing so, injury prevention programmes could be applied, and can ultimately lead to improved individual and team performances. Ideally, isokinetic strength, neuromuscular coordination, and ankle ROM should be evaluated, addressed and/or improved prior to the commencement of a netball season.

## Conclusion

The results indicated acute differences in the associations between ankle ROM, ankle joint muscle isokinetic strength, and lower extremity muscle activity amplitude as a function of lower extremity dominance and landing direction. Furthermore, the mean differences in EMG amplitude were not significantly different across landing limb or landing direction, potentially indicating similar neuromuscular strategies. There was insufficient evidence to conclude that lower extremity dominance was a predisposing factor for injury, and that specific joints were more susceptible to injury compared to others. However, injuries to the ankle and back were generally reported with higher prevalence in this cohort of netball players.

## Figures and Tables

**Table 1:** Within subjects’ effects for EMG amplitude during the eccentric phase of landing

	Sum of squares	df	Mean squares	F value	P value	η^2^p
Leg	17794	1	17794	0.93	0.360	0.09
Residual	172499	9	19166			
Direction	120585	4	30146	1.03	0.406	0.10
Residual	1055801	36	29328			
Muscle	973457	2	486728	16.84	< 0.001	0.65
Residual	520378	18	28910			
Leg ✻ Direction	33120	4	8280	0.57	0.683	0.06
Residual	519630	36	14434			
Leg ✻ Muscle	9730	2	4865	0.26	0.772	0.03
Residual	333593	18	18533			
Direction ✻ Muscle	181791	8	22724	1.94	0.067	0.18
Residual	844418	72	11728			
Leg ✻ Direction ✻ Muscle	42490	8	5311	1.13	0.353	0.11
Residual	337960	72	4694			

✻ interaction between variables
